# Transcriptomic analysis reveals candidate molecular pathways involved in pea (*Pisum sativum* L.) resistance to pea aphid (*Acyrthosiphon pisum* Harris) biotypes

**DOI:** 10.1186/s12864-025-11742-3

**Published:** 2025-07-01

**Authors:** Rémi Ollivier, Stéphanie Robin, Marc Galland, Po-Yuan Shih, Stéphanie Morlière, Maria K. Paulmann, Jonathan Gershenzon, Grit Kunert, Marie-Laure Pilet-Nayel, Jean-Christophe Simon, Akiko Sugio

**Affiliations:** 1 IGEPP, INRAE, Institut Agro, Univ Rennes 1, Le Rheu, 35653 France; 2 Present Address: Center for Quantitative Genetics and Genomics, Aarhus University, Forsøgsvej 1, Slagelse, 4200 Denmark; 3 INRIA, CNRS, IRISA, Rennes, 35000 France; 4 Present Address: Department of Life Sciences, Imperial College London, London, SW7 2AZ UK; 5 Department of Biochemistry, Max-Planck Institute for Chemical Ecology, Jena, Germany

**Keywords:** *Pisum sativum* L., *Acyrthosiphon pisum* Harris, Plant-aphid interaction, Molecular response, Aphid resistance, Transcriptomics, Plant defense mechanisms, Gene expression

## Abstract

Pea (*Pisum sativum* L.) is a major legume crop frequently infested by various parasites, including the pea aphid (*Acyrthosiphon pisum* Harris), which takes nutrients from its host and transmits phytopathogenic viruses. This aphid species comprises several biotypes, each one capable of feeding and reproducing on a limited range of specific legume species. However, little is known about the transcriptional response of the plants to different aphid biotypes. To investigate pea defense mechanisms against the pea-adapted and pea non-adapted *A. pisum* biotypes, we analyzed the transcriptional responses by mRNA sequencing of six pea genotypes with contrasting resistance levels and different haplotypes at the *ApRVII* locus. This locus confers partial resistance to the pea-adapted and non-adapted *A. pisum* biotypes. Transcriptomic analyses of pea genotypes with and without aphid infestation revealed 9,217 differentially expressed genes in pea genotypes infested with the pea-adapted aphids, while fewer genes, 1,561 genes total, were expressed in response to the pea non-adapted aphids. Both aphid biotypes activated immune responses and the biosynthesis of secondary metabolites, including flavonoids. However, the pea-adapted biotype appeared to suppress multiple pathways associated with photosynthesis, cell wall biosynthesis, fatty acid metabolism, and other growth-related processes. Candidate genes potentially involved in aphid resistance were identified both within and outside of the *ApRVII* locus. These findings provide insights into pea resistance mechanisms against both pea-adapted and pea non-adapted *A. pisum* biotypes, as well as the ability of the adapted biotype to modulate pea defenses leading to host susceptibility, and pave the way for follow-up studies, including metabolomic analyses.

**Supplementary Information:**

The online version contains supplementary material available at 10.1186/s12864-025-11742-3.

## Introduction

Insect pests reduce crop yields by taking up nutrients from the plant and by transmitting plant pathogens. In conventional agriculture, pesticides have been used to control them, but there is growing concern about their harmful effects on human health and the environment [1]. Aphids are insect pests that feed on the plant’s phloem sap and cause considerable damage directly by feeding and indirectly by transmitting plant pathogenic viruses. During feeding, aphids insert their mouthparts to gain access to the phloem and release saliva to facilitate stylet insertion as well as to disrupt the biological functions of the plant. Many aphid species are specialised to develop on a few plant species, while a few others are more generalist and feed on a wide range of plant families [2]. In order to control aphid damage without relying on pesticides, it is important to understand the mechanisms of plant resistance or susceptibility to aphids.

Plant-aphid interactions and the mechanisms determining their compatibility and incompatibility can be explained using the paradigm developed for plant-microbial pathogen interactions. Aphids secrete saliva containing numerous proteins into plant cells [3]. These proteins, originating from both the aphids and their endosymbionts, can act as herbivore-associated molecular patterns (HAMPs), also known as elicitors, triggering plant defense reactions. For instance, GroEL is an aphid HAMP produced by the aphid’s primary bacterial endosymbiont, *Buchnera aphidicola* Munson [4]. Found in aphid watery saliva, GroEL induces pattern-triggered immunity (PTI) in tomato [5]. Additionally, some components of aphid saliva, known as effectors, are shown to suppress plant defense and others may trigger the defense reaction. However, the modes of action of most aphid effectors remain largely uncharacterized [2, 6, 7]. This lack of knowledge is not limited to aphid effectors: the plant genes responsible for recognizing HAMPs or those targeted by aphid effectors are also poorly understood, despite their importance in determining the outcome of plant-aphid interactions.

To identify the plant responses during infestation with adapted and non-adapted aphids (host and poor- or non-host interactions, respectively), two microarray-based studies were conducted [8, 9]. The first study showed the transcriptomic changes occurring in *Arabidopsis thaliana* (L.) Hevnh infested by *Myzus persicae* Sulzer (host interaction), *Myzus cerasi* Fabricius (poor-host interaction) and *Rhopalosiphum padi* L. (non-host interaction) [8]; while the second study showed transcriptome changes of *Brassica juncea* (L.) Czern infested by *Lipaphis erysimi* Kaltenbach (host-interaction) or *Aphis craccivora* Koch (non-host interaction) [9]. Both studies showed common and specific plant responses during the host and non-host interactions. More differentially expressed genes (DEGs) compared to non-infested plants were identified during the host interactions than non-host interactions. In addition, most of the DEGs identified in the host interaction and overlapping with non-host interaction showed the same sense (up or down) of regulation, indicating the induction of common responses, likely triggered by the perception of the HAMPs. Other transcriptomic studies have examined plant species with or without major aphid resistance genes, which lead to compatible or incompatible interactions with aphids [10, 11]. Le Boulch et al. examined the transcriptome of peach (*Prunus persica* (L.) Batsch) genotypes infested with *M. persicae* [10]. A higher number of DEGs was induced in aphid resistant genotype carrying *Rm2* resistance gene than the susceptible one. The resistant genotype induced a massive defence reaction and suppression of genes involved in cell growth and cell division was observed [10]. MacWilliams et al. (2023) studied resistant and susceptible near-isogenic lines (NILs) of cowpea (*Vigna unguiculata* (L.) Walp.) infested with the cowpea aphid (*A. craccivora*) [11]. Similar number of DEGs were obtained during compatible and incompatible interactions. However, the transcriptional reprogramming occurred more slowly in the resistant NIL infested by the aphid compared to the susceptible NIL. DEGs potentially involved in aphid resistance were found in two regions previously identified as QTLs for aphid resistance.

These studies have shown that each plant-aphid interaction induces specific responses of the plant to the aphids, depending on the species studied, suggesting that the plant pathways involved in defense against adapted and non-adapted aphids vary and cannot be generalized. Hence, it is necessary to build knowledge on the plant-aphid interactions of scientific and agronomic interest. Among plant species of interest, pea (*Pisum sativum* L.) is a cultivated crop belonging to the *Fabaceae* family that offers environmental, agronomic and economic advantages [12, 13]. Pea can be used to diversify crop rotations, while limiting nitrogen inputs due to its nitrogen-fixing symbiosis and producing relatively high amounts of protein. Unfortunately, this crop is subject to high levels of biotic stress in the field, particularly from the pea aphid (*Acyrthosiphon pisum* Harris), which takes nutrients from its host and transmits phytopathogenic viruses [14–16]. The pea aphid is a complex of at least 15 genetically different biotypes, each specialized to feed and develop on one specific or a few legume species [17, 18]. A study of feeding behavior of different biotypes on varying legume species showed, that the feeding duration and ability of the aphid is influenced by host or non-host interactions [19]. Further, observation of pea-adapted and alfalfa-adapted *A. pisum* biotypes on five *P. sativum* genotypes showed that the alfalfa-adapted biotype spent shorter amount of time feeding from the phloem sap compared to the pea-adapted biotype, indicating that the non-adapted biotype’s feeding activity is impaired at the mesophyll and phloem cell level [20].

Pea resistance against different *A. pisum* clones of pea-adapted and pea non-adapted (alfalfa-adapted) biotypes was assessed by aphid performance on the pea Architecture and Multi-Stress (AMS) collection containing 240 pea genotypes [21]. The results indicated that the pea-adapted biotype reproduced on all pea genotypes, but some pea genotypes displayed partial resistance compared to other susceptible pea genotypes. Most of the pea genotypes were highly resistant to the pea non-adapted biotype, but some exhibited partial susceptibility. To identify the genetic bases underlying partial resistance to pea-adapted and high resistance to pea non-adapted aphids, we performed a genome-wide association study (GWAS) using the phenotypic data and genotypic data generated by exome sequencing. The *ApRVII* region, spanning chromosome 7 between 129 Mb and 161 Mb, was identified as the locus controlling the pea resistance against pea-adapted and pea non-adapted biotypes of *A. pisum* [21]. However, the molecular mechanisms related to this locus are not understood. The genotyping data used for the GWAS were obtained by exome capture and some candidate genes underlying *ApRVII* were identified as containing resistance-associated SNPs in their sequences. The causal variant can be located either in a coding region or in a non-coding region. A mutation located in a non-coding region could affect the expression of one or some genes responsible for resistance, while a mutation in a coding region could affect a gene function.

Resistance to different *A. pisum* biotypes was also studied in *Medicago truncatula* Gaertn. and *RAP1* locus which confers resistance to a *A. pisum* pea-adapted biotype clone [22] and the candidate salivary genes that may function as virulence and avirulence factors were identified [7, 23]. However, the molecular defense response of *M. truncatula* to *A. pisum* is still not fully unraveled.

In the presented study, we analyzed the pea transcriptional response to pea-adapted and pea non-adapted aphids to identify genes or pathways associated with the resistance and susceptibility to the aphids. Six genetically close pea genotypes, with different haplotypic sequences at the *ApRVII* locus and contrasting levels of resistance against pea-adapted and non-pea-adapted *A. pisum* biotypes were used for the study [21]. First, we aimed to have an insight into the general pea response to infestation with two biotypes of *A. pisum*. The global pea transcriptomic response of the selected pea genotypes was investigated during host and non-host interaction. We hypothesized that distinct genes and pathways may be differentially expressed in the two interactions. Second, we aimed to identify the genes that may be involved in the pea resistance mechanisms to *A. pisum* biotypes, especially involving *ApRVII.* We hypothesized that the genes involved in aphid resistance would show distinct expression patterns associated with the resistant phenotype.

## Materials and methods

### Plant material

The six pea genotypes were selected from the pea AMS collection [24] for their resistance and susceptibility to *A. pisum* pea-adapted and pea non-adapted biotypes [21]. These pea genotypes were AeD99QU-04-4-6-1 (hereafter: AeD1); AeD99OSW-50-2-5 (hereafter: AeD2); AeD99OSW-49-5-7 (hereafter: AeD4); CE101 = FP (hereafter: FP) and KAZAR. The seeds of AeD3 were stored as AeD99OSW-55-6-4. However, genotype-by-sequencing analysis described below showed that AeD3 is highly similar to the pea genotype AeD99OSW-56-19-4 [21].

The pea genotypes AeD2, AeD4 and KAZAR were partially resistant to the pea-adapted biotype ArPo28, while AeD1, AeD99OSW-55-6-4 and FP were susceptible. AeD2, AeD4 and KAZAR were highly resistant against the non-adapted biotype LSR1, while AeD1, AeD99OSW-55-6-4 and FP were partially susceptible [21].

The six pea genotypes belong to two distinct genetic subgroups. The first genetic subgroup includes AeD1, AeD2, AeD3 and AeD4. These four pea genotypes are spring-sown genotypes coming from Aphanomyces root rot breeding programs (Table S1) [21, 25]. The second genetic subgroup contains FP and KAZAR that are winter-sown genotypes (Table S1) [21, 25]. Haplotype sequences underlying the *ApRVII* locus for these pea genotypes were identified in the previous study [21] and are reported in Table S1. The pea plants were grown in pots (one plant per pot of 7 × 7 × 8 cm) in a climatic chamber set at 18 ± 1.5 °C and 16 h day/8 h night cycle. Two-week-old plants were used for fecundity assays and aphid infestation assays for RNA sequencing.

### Insect material

Aphids were reared in a long day growth chamber (18 ± 1.5 °C, 16 h day/8 h night cycle) to maintain parthenogenetic reproduction. Aphids were reared on *Vicia faba*, which is a common host for all the biotypes of *A. pisum* [26]. We used the P123 clone, which is adapted to feed on pea (pea biotype) and the LSR1 clone, which is adapted to alfalfa (alfalfa biotype) to infest the pea plants [21]. P123 clone was used to replace the clone ArPo28, which was used for previous study but lost after. Both aphid clones do not carry any secondary bacterial symbiont. Note that the fecundity tests of ArPo28 and P123 on six pea genotypes showed similar results [21].

### Fecundity assays

The six genotypes of *P. sativum* plants were grown in pots (one plant per pot of 7 × 7 × 8 cm) in a climatic chamber, set at 18 ± 1.5 °C and 16 h day/8 h night cycle. To remove potential maternal effects of feeding on a universal host (*V. faba*), P123 aphids were reared on the test pea genotype for two generations before assessing the fecundity. Ten days after the installation of a one-day-old larva (G1), a single one-day-old larva from the second generation (G2) was installed on one new *P. sativum* plant of the same pea genotype. Then, 15 days after the installation of G2 aphids, all their offspring (G3) were collected and counted. To assess the fecundity of the LSR1 clone, a different protocol was applied. A single one-day-old larva produced on *V. faba* was installed on each *P. sativum* genotype and all aphids (G2 offspring) were collected and counted three weeks later. The fecundity tests for P123 were carried out in two independent experiments and once for LSR1. In both assays, 20 to 30 replicates (one plant per replicate) per *P. sativum* genotype were used per test. Two to three plants were randomly chosen from each of the six pea genotypes and placed on the same tray (used to hold the plants in the growth chamber, one tray containing a maximum of 35 plants). The potential impact of the tray placement was accounted for in the statistical analysis.

### Statistical analysis of phenotypic data

All statistical analyses were conducted with R version 4.3.1 [27]. A Generalized Linear Mixed Model (GLMM) integrating a negative binomial parameterization [R package “glmmTMB” version 1.1.10 [28]] was applied to analyze the number of aphid offspring from the infestation with the pea-adapted clone P123. In the model, the pea genotype was used as fixed factor and “Experiment” (replicate of the fecundity assessment) and “Tray” as random factors in the model. For the analysis of the pea non-adapted LSR1 fecundity data, we executed a model based on a zero-inflated generalized Poisson parameterization. This model incorporated the “Tray” as a random effect and the “Genotype” as fixed explanatory variable. For completely resistant genotypes to LSR1, we deliberately modified one of the “0” values to “1” to introduce minimal variability for this factor level. This adjustment was necessary because without any variability, the standard error for this factor level would be extremely large and essentially meaningless. By introducing this minimal variability, we achieved a more realistic estimation of the standard error. Post-hoc comparisons were performed using the emmeans package version 1.10.4 [29] to calculate Estimated Marginal Means (EMMs) for each pea genotype. Pairwise comparisons of EMM values with Tukey’s adjustment for multiple testing (α = 5%) was applied using the “lsmeans” function of the R package emmeans version 1.10.4 [29].

### Sample Preparation for RNAseq analysis

Each two-week-old pea plant was infested with 100 three-day-old aphids caged by a perforated plastic bag and maintained in a climatic chamber set at 18 ± 1.5 °C, 16 h day/8 h night cycle. The aerial parts of the plants were cut at 72 and 120 h post infestation (hpi) and flash frozen in liquid nitrogen. All the aphids on the frozen plants were removed by a paint brush. Non-infested plants were treated in the same manner and served as controls for each time point. Plant samples for RNA extraction were grounded with liquid nitrogen using mortars and pestles. Non-infested plants were used as control samples for both time points. Three biological replicates were prepared for each infestation condition, resulting in a total of 108 samples for RNA sequencing.

### Transcriptomic analysis

Total RNA was extracted with the RNeasy Plant Mini Kit using the QIAZOL lysis reagent (QIAGEN), and DNA was treated with an RNase-Free DNase Set (QIAGEN). RNA and DNA were quantified by using a Quantus fluorometer (QuantiFluor), and RNA quality was assessed using a 2100 Bioanalyzer (Agilent Technologies). Poly-A enrichment from total RNA and library preparation was carried out by Genewiz (Leipzig, Germany), and the RNA sequencing was performed on an Illumina NovaSeq to generate 150-nucleotide-long paired-end reads. The raw data pre-processing was conducted using the nextflow RNAseq pipeline version 3.4 [30], by including the parameters “--stringtie_ignore_gtf” to perform a reference-guided de novo assembly of transcripts using StringTie and “--remove_ribo_rna” to enable the removal of reads derived from ribosomal RNA using SortMeRNA [31]. To assess the sequence quality of the raw data, FastQC software version 0.11.9 was used [32]. Data were trimmed for low-quality and adapter sequences using TrimGalore version 0.6.7. The ribosomal RNAs fragments were removed with sortMeRNA version 4.3.4. Once filtered, the reads were aligned against the Pea Reference Genome sequence v1a [33] using the HISAT2 v2.0.5 alignment program [34]. StringTie version 2.1.7 was used to assemble the alignments into potential transcripts for each gene locus [35]. All the previously described steps from quality control of raw sequencing data to annotation of new transcripts with Stringtie were conducted with the nextflow RNAseq pipeline [30, 31]. Long non-protein-coding transcripts were identified using the FEElnc v0.1.0 software [36] with default parameters. Mapped reads were assigned to the pea reference genome v1a [33] enriched with the new transcripts using FeatureCounts V2.0.1 [37]. Correctly and uniquely mapped reads on the same chromosome were counted using the featureCounts tool with the corresponding flags (-B, -C, -p, -s 2). The -B option ensures that both ends of paired-end reads are mapped to the same chromosome and strand, and that the distance between them falls within a specified range. The -C option disables the requirement that reads must be fully contained within feature boundaries. The -p option specifies that pairs of reads should be counted instead of individual reads, which is particularly useful for paired-end sequencing data. The -s 2 option indicates that the reads are reversely stranded.

### Annotation of protein-coding genes

Protein-coding transcripts, obtained from FEElnc v0.1.0, were predicted using TransDecoder version v5.5.0 [38], to predict ORFs of at least 50 amino acids longs, including the optional homology searches based on BlastP and Pfam domain search (BlastP using BLAST version 2.9.0+ [39] and Pfam domain search using HMMER version 3.3 [40]). On the whole proteome, we performed the functional annotation using EggNOG mapper v.2.1.6 with EggNOG database v.5.0.2 [41, 42] and InterProScan5 version 5.54-87.0 [43]. GO term annotation was obtained in EggNOG outputs. When a given gene is annotated with more than one predicted ORF, only the function of the first ORF prediction was used to annotate the corresponding transcript.

KEGG annotation was obtained using the annotated *M. truncatula* genome V5 [44]. To identify the corresponding genes all coding DNA sequences (CDS) from the pea genome Cameor [33] were blasted on the CDS of the *M. truncatula* genome V5 [44] using BlastN version 2.9.0+ [39]. The blast consisted of aligning the CDS sequences of both genomes with an e-value < 0.001 and the best hit was conserved.

### Statistical analysis of RNAseq data

The principal component analysis (PCA) was performed using the RNA-seq count data, following the procedure described in [45] and a custom R function. To stabilize the variance, the R package DESeq2 version 1.40.2 was used. Size factors and dispersions were estimated, and a variance-stabilizing transformation was applied. A custom function was developed to perform PCA using singular value decomposition (SVD) with the base R package version 4.3.1. SVD was applied to the reduced matrix, which was centered and scaled using the R function scale. All the analyses of differential gene expression, clustering analyses and GO enrichment analyses described in this section were performed using the AskoR pipeline [46, 47]. Differential expression between control and infested samples was obtained using the R package edgeR version 3.42.4 [48]. Genes expressed with a CPM > 1 for at least five samples were conserved for the differential expression analyses. Genes were considered as differentially expressed when the false discovery rate (FDR)-adjusted *p*-value was less than 0.05 and the fold change was higher than 1.5.

To visually compare multiple lists of genes, we used the upset graph representation proposed in the AskoR pipeline with the R package UpSetR version 1.4.0. To visually compare the number of differentially expressed genes between pea genotypes and in all the conditions of infestation, we used barplots produced with the R package ggplot2 version 3.5.1.

Co-expression analysis of the DEGs was conducted using coseq R package version 1.24.0 [49, 50] included in AskoR pipeline using the “ExpressionProfiles” approach (sample expression / sum of expression in all samples) with “clr” (centered log-ratio) transformation and a “kmeans” clustering. By default, coseq choose a number between 2 and 25 clusters based on the slope heuristics. GO term enrichment analysis was conducted using the TopGO R package version 2.34.0 [51], with the weight01 algorithm and Fisher test. The analysis included a selection of GO terms containing at least ten DEGs and a significant/expected ratio of more than 2. Adjusted *p*-values for these GO terms were obtained with the FDR method, using the R function p.adjust from the package stats. Enrichment analysis of the KEGG pathways was conducted using the R package clusterProfiler version 4.8.3 using the FDR *p*-value cutoff of 0.05 [52].

The EdgeR tool allows for the use of models incorporating fixed variables to identify differentially expressed genes. However, to account for the confounding effect of genotype-specific expression patterns in the identification of genes involved in resistance, it was necessary to include genotype as a random variable in the model. EdgeR was used for the analysis of DEGs between plants infested and non-infested with aphids, while the glmmSeq tool [53] was employed for the analysis of DEGs between resistant and susceptible genotypes across all infestation conditions. In the glmmSeq model, we included several fixed effects: the “resistance” factor according to the qualitative resistance levels of the pea genotypes against one of the two aphid biotypes (P123 or LSR1), the “timepoint” to include the two timepoints (72 h and 120 h), the “infestation” to have the difference between non-infested and infested with one of the two aphid biotypes (P123 or LSR1), and all the interactions between these three variables indicated with the term “:”. A Generalized Linear Mixed Model (GLMM) integrating a negative binomial parameterization [R package “glmmTMB”] was applied to integrate the previously described fixed effects but also to integrate the “genotype” as random variable in the model. The analysis primarily focused on the FDR-adjusted *p*-values associated with resistance, as well as the interactions between resistance and either condition, timepoint, or both variables. The model was written as below:

glmmSeq (Gene_expression ~ Resistance * Timepoint * Infestation + (1|Genotype), method = c(“glmmTMB”)).

### Identification of the pea genotype AeD3

The phenotyping assay suggested that the AeD3 was not the original genotype AeD99OSW-55-6-4. To resolve the identity of the pea genotype AeD3, we used one of the alignment files previously produced from a non-infested AeD3 sample, mapped on the reference genome Caméor [33]. To reduce the computing power required, only alignments on the chromosome 7 containing the *ApRVII* locus were filtered using samtools version 0.1.18 with the samtools view command [54]. A SNP calling was conducted using GATK version 4.3.0.0 with the HaplotypeCaller function (parameters: --standard-min-confidence-threshold-fo-calling 20, --dont-use-soft-clipped-bases) and using the same reference genome [33]. A quality filtering was applied using bcftools version 1.9 [54] to filter SNPs with quality QUAL > 30 and read depth INFO/DP > 10. A normalization was applied using bcftools norm command. Both VCF files, one containing the SNP information of AeD3 and the second containing SNP information of the whole AMS collection, were merged in a single VCF file with bcftools merge command, and the parameter “--force-samples” to conserve all the SNPs. To calculate the identity-by-state distance between AeD3 and all the other pea genotypes from the AMS collection, we used Plink version v1.9 with the parameter “--genome” [55]. We also used the same procedure for the five other pea genotypes, as controls.

## Results

### Six pea genotypes showed contrasted resistance phenotype

**Fig. 1:**
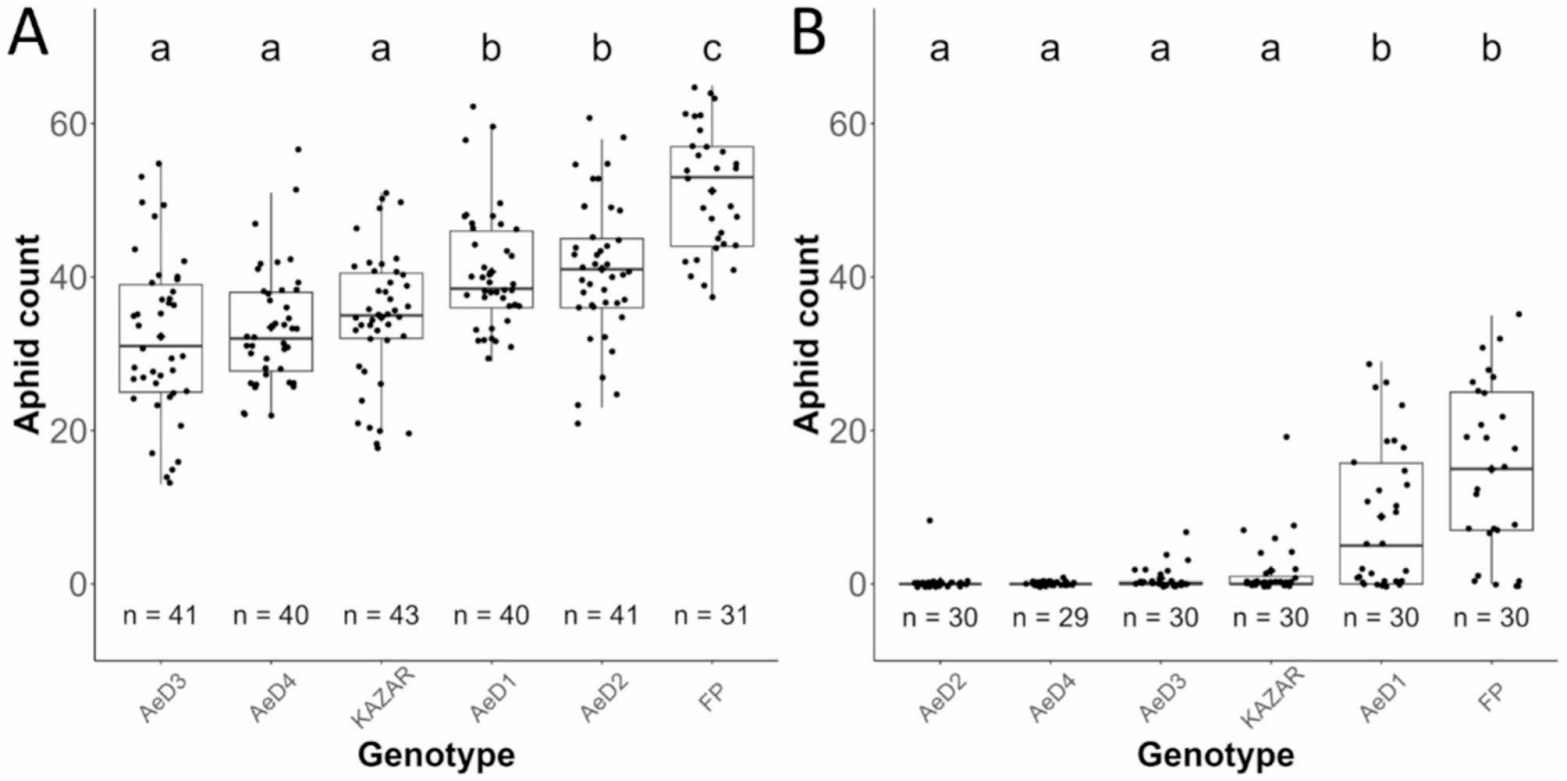
Aphid fecundity (aphid count) on the six pea genotypes. Fecundity assays of the pea adapted P123 (**A**) and the non-adapted LSR1 (**B**) clones showed a contrasted resistant phenotype of the pea genotypes. Different letters (**a**, **b**, **c**) indicate significant statistical differences based on pairwise comparisons of Estimated Marginal Means (EMMs) with Tukey’s adjustment for multiple testing (α = 5%). The number of biological replicates for each pea genotype is shown with “n=”

Six pea genotypes were selected among the pea Architecture and Multi-Stress (AMS) collection for (1) their contrasted levels of resistance to ArPo28 (pea biotype) and LSR1 (alfalfa biotype), (2) their genetic relatedness (to minimize the effect of genetic distance in comparisons) and (3) their various haplotype sequences underlying the *ApRVII* locus [21, 25]. To verify the resistance levels of these six pea genotypes against P123 (pea biotype of *A. pisum*) and LSR1 (alfalfa biotype of *A. pisum*), we conducted a new fecundity test. For the resistance to P123, the pea genotypes AeD3, AeD4 and KAZAR were partially resistant while AeD1, AeD2 and FP were susceptible (Fig. 1A). AeD2, AeD3, AeD4 and KAZAR were highly resistant against LSR1, while AeD1 and FP were partially susceptible (Fig. 1B). The phenotyping results for the genotypes AeD1, AeD4, KAZAR and FP are consistent with the results obtained in [21]. The genotype AeD2 was partially resistant to ArPo28 (pea biotype) in the previous study but was found to be susceptible to P123 (pea biotype) in this study. The results of both studies were consistent regarding the resistance of AeD2 to LSR1. AeD3 was partially resistant to P123 (pea biotype) and resistant to LSR1 (alfalfa biotype) (Fig. 1), which contradicted the previous fecundity results of the genotype AeD99OSW-55-6-4 being susceptible to both aphid clones. The genotype-by-sequencing analysis of AeD3 showed that it carried haplotypes associated with the resistance to ArPo28 (pea biotype) and LSR1 (alfalfa biotype) under the locus *ApRVII* (Table S1). The haplotypes were very similar to the pea genotype AeD99OSW-56-19-4 used in the previous study (Table S1) [21] and the results of the fecundity assay supported that. Thus, we concluded that AeD3 is most likely AeD99OSW-56-19-4 and not AeD99OSW-55-6-4 as initially planned.

### Pea-adapted aphids induced massive transcriptomic reprogramming in pea while pea non-adapted aphids induced weak stress responses

**Fig. 2:**
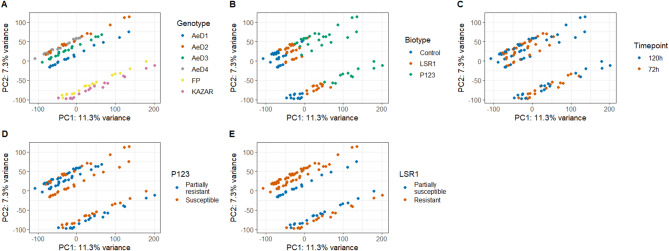
Principal component analysis (PCA) of the gene expression data. PCA based on the gene expression data of 108 libraries of pea genotypes infested or not with two pea aphid biotypes. The two first dimensions are displayed in this figure. The samples are coloured according to (**A**) the pea genotypes (**B**) the conditions of infestation, (**C**) the sampling timepoints, (**D**) the resistance to the pea-adapted biotype P123 and (**E**) the resistance to the non-adapted biotype LSR1

A total of 108 libraries were generated for RNA sequencing with a median of 19.7 million reads. The alignment on the reference genome provided an overall median alignment of 90.4%, and on average 86.4% of the reads were paired and aligned once (Table S2). Principal component analysis (PCA) resulted in a clear clustering of the samples by the pea genotypes, indicating that gene expression patterns are mostly specific to the genotype (Fig. 2A, D and E) and no clear association with aphid-resistance phenotypes. The PCA also showed, for each genotype, a clear separation between the control plants and the P123-infested plants, while the LSR1-infested plants were mostly located in between these two conditions of infestation (Fig. 2B). This observation suggested that the infestation with P123 triggered a stronger pea response compared to the infestation with LSR1. The samples of 72 and 120 hpi were not separated except for some samples infested with P123 (Fig. 2C).

**Fig. 3:**
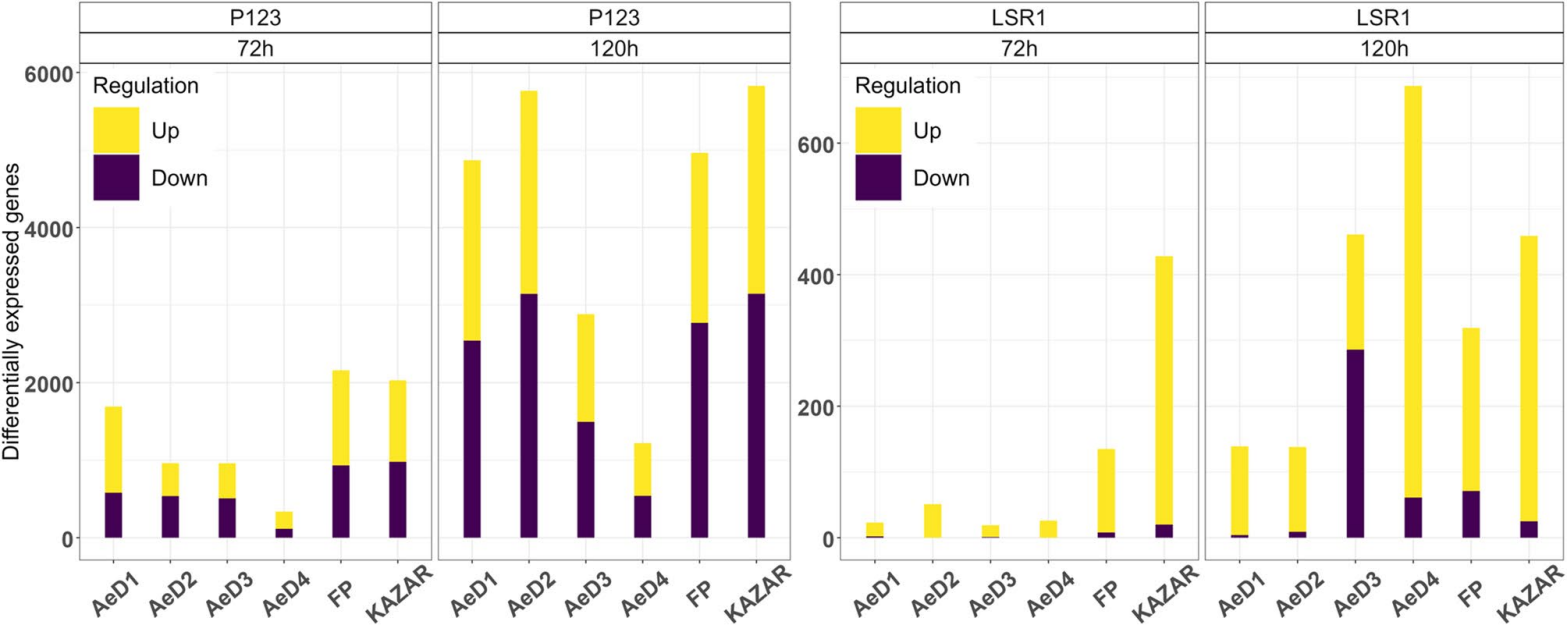
Number of differentially expressed genes in the pea genotypes infested with two pea aphid biotypes. The barplots display the number of up and down-regulated genes in the six pea genotypes infested with P123 and LSR1 for 72 h and 120 h. The number of differentially expressed genes increased from 72 to 120 hpi, regardless of the aphid clones and the pea genotypes. The pea response was stronger during infestation with P123 compared to the infestation with LSR1

We detected 21,008 genes that were expressed in at least five samples (CPM > 1) among the 108 samples and 9,484 differentially expressed genes (|FC| > 1.5) in at least one condition of infestation (Table S3, Table S4) compared to non-infested control. Among all DEGs, 9,217 were identified during the interaction with P123, while 1,561 DEGs were identified during the interaction with LSR1 (Table S3). Pea plants infested with the pea-adapted aphid clone, P123, induced a stronger response compared to the pea non-adapted clone, LSR1 (Figs. 2 and 3). In both interactions, the number of DEGs increased from 72 to 120 hpi. More DEGs were down-regulated during the infestation with P123 compared to the infestation with LSR1 (Fig. 3, Table S3 and S4).

During infestation with P123, a strong overlap between DEGs identified at 72 and 120 hpi was observed. Among the down-regulated genes, this overlap represented from 71.3% (pea genotype AeD4) to 95.53% (pea genotype AeD2) of all the DEGs identified at 72 hpi (Table S4). Among the up-regulated genes, this overlap represented from 65.31 to 85.61% of all the DEGs identified at 72 h (Table S4). The intersection analyses of the DEGs showed that a large part of the DEGs were genotype specific, but significant numbers of genes (177 and 714 DEGs at 72 and 120 hpi, respectively) were commonly up or down regulated in all the six pea genotypes (Figure S1).

These analyses also showed that the pea genotypes FP and KAZAR, which belong to the same genetic group, shared more group specific DEGs than the group of the four AeD genotypes. Indeed, at 72 hpi, 241 DEGs and a single DEG were identified in the FP/KAZAR genetic group and in the AeD group, respectively. At 120 hpi, 277 DEGs and 5 DEGs were respectively identified in the FP/KAZAR genetic group and in the AeD group (Fig. S1).

During the infestation with LSR1, the four AeD genotypes showed a lower number (19 to 51 DEGs) of DEGs at 72 hpi compared to the genotypes FP (135 DEGs) and KAZAR (428 DEGs) (Fig. 3 and Table S4). The responses increased at 120 hpi for all of the AeD genotypes, but were particularly strong for the genotypes AeD3 (461 DEGs) and AeD4 (687 DEGs) (Fig. 3 and Table S4). The majority of DEGs triggered by LSR1 infestation were up-regulated (70.1%) and were mostly genotype specific at 72 hpi (76.8%) and 120 hpi (71.9%) (Table S3). At 72 hpi and 120 hpi, 48 DEGs and 17 DEGs were specifically identified in the FP/KAZAR genotypes, but not in the AeD group and no DEG was commonly and specifically identified in the AeD group. Only two and 15 DEGs were respectively identified at 72 and 120 hpi as commonly regulated in the six pea genotypes (Figure S1).

Most of the DEGs identified during the infestation with LSR1 were also identified during the infestation with P123 in each pea genotype (Figure S2). About 82.9% (1,294 DEGs) of all the DEGs (1,561 DEGs) detected in the response to LSR1 were shared with the DEGs identified during the interactions with P123 (Table S3, Figure S2). Most of the DEGs identified during the infestation with P123 and LSR1 showed the same direction of regulation. Only five DEGs (*Psat0ss5882g0160.1*, *Psat1g122080.1*, *Psat6g154120.1*, *Psat7g248600.1* and *MSTRG2.13096.2*) showed the opposite direction of regulation between the infestation with P123 and LSR1. Four were identified in the pea genotype KAZAR and one in the pea genotype AeD1 (Table S3).

### General pea response to the infestation with pea-adapted and non-adapted aphid clones

**Fig. 4:**
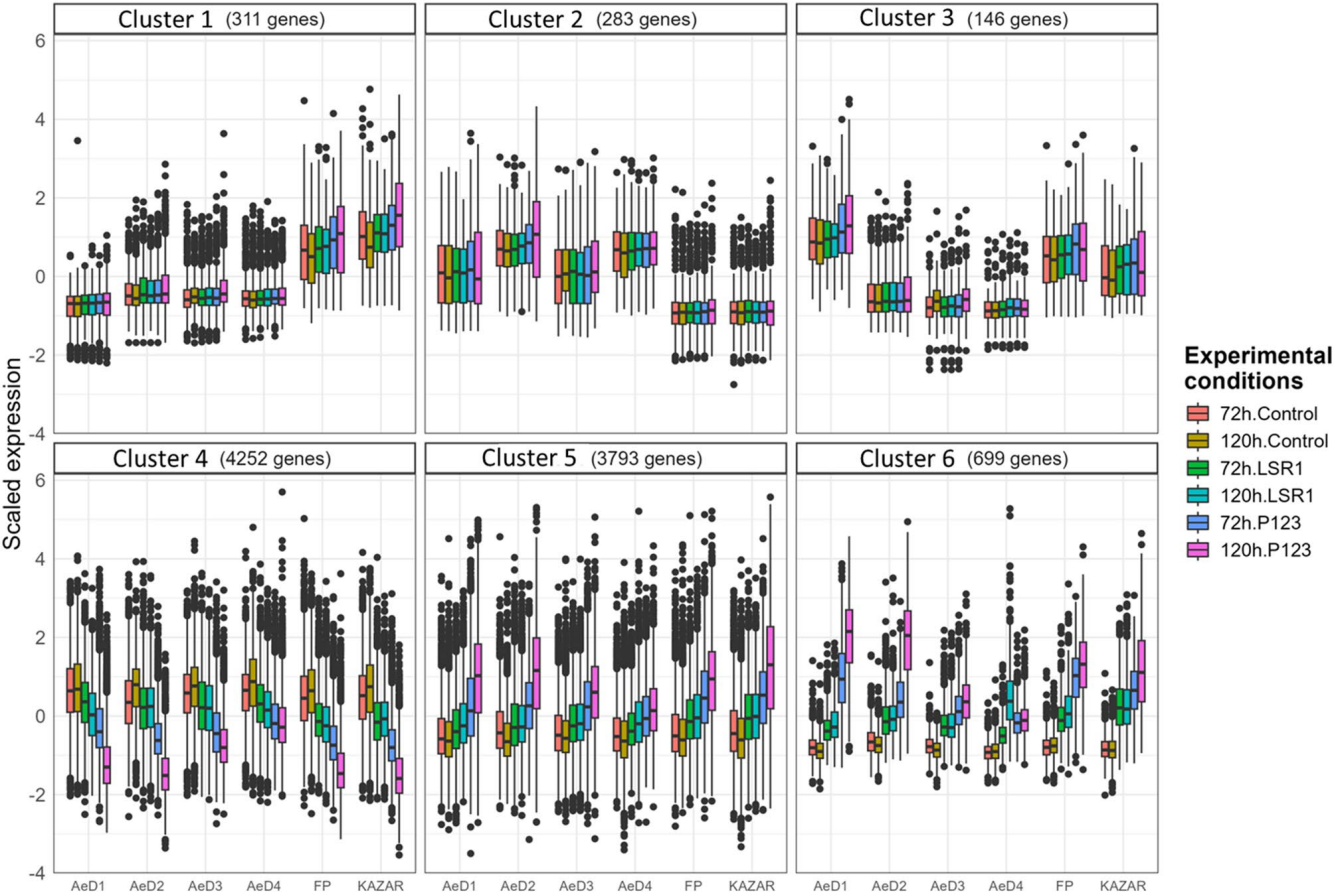
Boxplots of log-scaled DEG expression levels during aphid infestation, clustered into six groups. Boxplots displaying the log-scaled expression level of differentially expressed genes in six pea genotypes during aphid infestation, and clustered into six groups according to their expression patterns. Colors of the boxes indicate experimental conditions

To analyse the overall expression patterns of the 9484 DEGs identified under different infestation conditions and for each pea genotype, we conducted a clustering analysis. Six prominent clusters were identified based on the gene expression patterns (Fig. 4). Cluster 1 and 2 showed genes with genetic group specific (FP/KAZAR and AeD) expression levels (Fig. 4). These two clusters comprised 311 genes and 283 genes, respectively, that were more or less expressed in the FP/KAZAR group compared to the AeD group, regardless of the infestation condition. Cluster 3 consisted of 146 genes that were more expressed in the pea genotypes FP, KAZAR and AeD1 compared to the pea genotypes AeD2, AeD3 and AeD4, regardless of the infestation condition (Fig. 4). No GO term or KEGG pathway was significantly over-represented for these three clusters (Table S5).

**Fig. 5:**
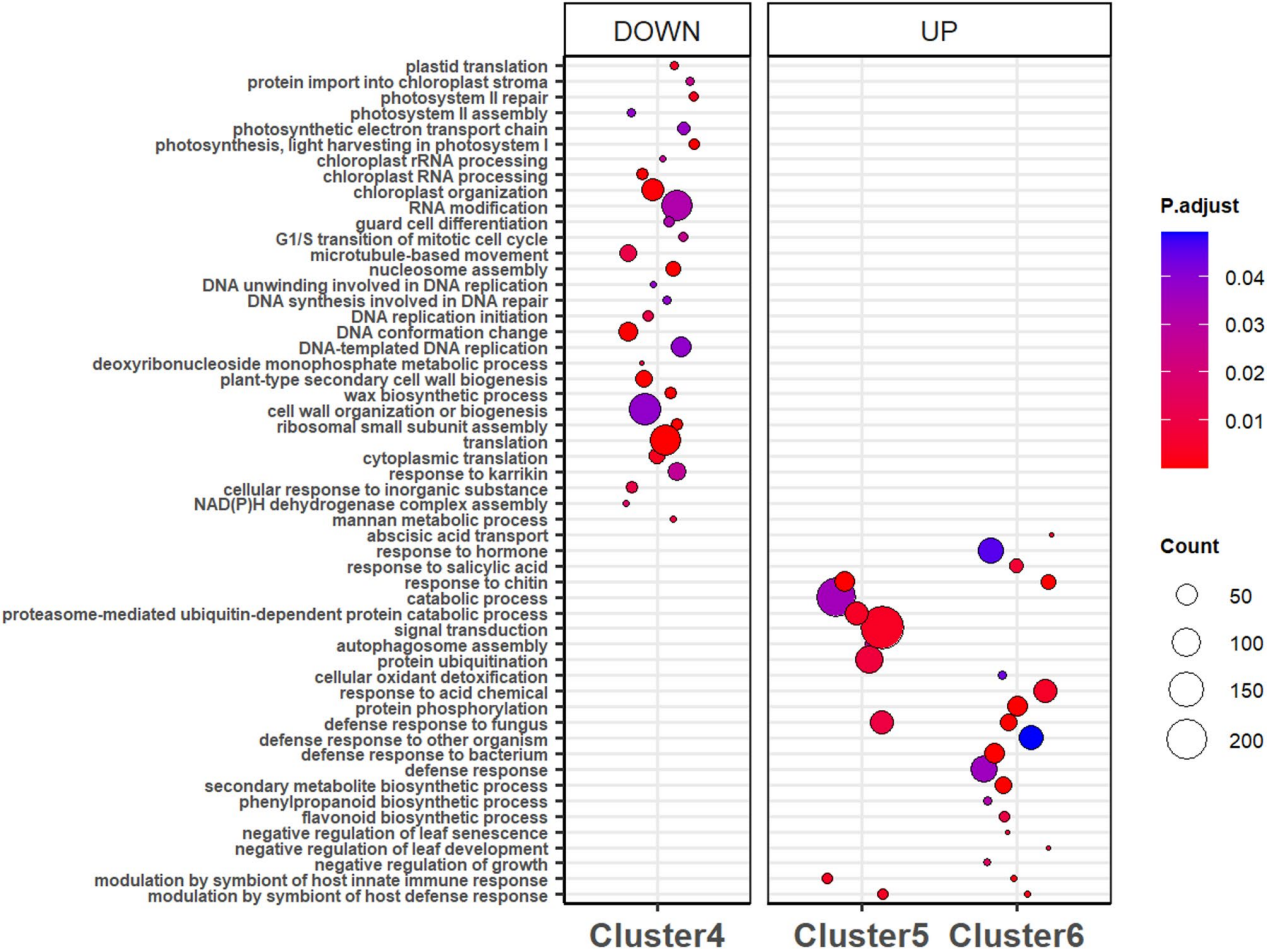
Dot plot of GO biological processes in response to aphid infestation. GO terms enriched in the gene set constituting clusters 4, 5 and 6. Adjusted *p*-values (P. adjust) are shown by different colors and the number of genes (count) are shown by the size of the circles

Clusters 4 and 5 included 4252 down-regulated genes and 3793 up-regulated genes, respectively, by P123 infestation compared to control samples (Fig. 4). The genes tended to be more differentially regulated at 120 hpi than at 72 hpi, although the direction of the regulation was the same. In addition, the genes tended to be also slightly up or down-regulated in the LSR1-infested samples (Fig. 4). The strength of the gene regulation in these clusters was higher for the pea-adapted P123 than for the non-adapted LSR1 aphid, especially at 120 hpi. GO enrichment analysis of cluster 4 showed GO terms related to translation processes, RNA modification, general plant growth and cell wall organization, photosynthesis and cell division (Fig. 5, Table S5). KEGG pathway analysis of cluster 4 showed numerous pathways related to translation, DNA replication, photosynthesis, plant growth, and fatty acids metabolism (Table S6).

GO enrichment analysis of cluster 5 showed GO terms related to biotic stress such as “response to chitin”, “defense response to fungus” and “modulation of host defense response by symbiont”. Other GO terms were related to the protein ubiquitination and catabolic processes and signal transduction (Fig. 5, Table S5). KEGG pathway analysis of cluster 5 showed the over-representation of genes related to glycolysis, gluconeogenesis and galactose metabolisms, MAPK signalling (Table S6).

Cluster 6 encompassed 699 genes characterized by a strong up-regulation of gene expression upon infestation with P123 and LSR1 (to a lesser extent) compared to control samples (Fig. 4). Clusters 5 and 6 showed similar expression patterns and shared some enriched GO terms such as “defense response”, “defense response to bacterium”, “defense response to fungus”, “defense response to other organism”, “response to chitin” and “modulation of host defense responses by symbiont” (Fig. 5). GO enrichment analysis of cluster 6 also showed significant terms associated with the phytohormone signalling, especially for salicylic acid and abscisic acid transport. GO terms related to the biosynthesis of secondary metabolites and more specifically the biosynthesis of flavonoids and phenylpropanoids were enriched. Three GO terms suggested negative regulation of growth, leaf development and senescence (Fig. 5, Table S5). KEGG pathway analysis of cluster 6 revealed the over-representation of genes related to the biosynthesis of phenylpropanoids and more specifically flavonoids and isoflavonoids, the biosynthesis of alkaloids, related to the plant pathogen interaction, MAPK signalling (Table S6).

### Molecular pathways were differentially regulated between resistant and susceptible pea genotypes

To investigate the molecular pathways that were differentially regulated between resistant and susceptible pea genotypes upon aphid infestation, all 21,008 expressed genes in the six pea genotypes were analyzed for their expression in all conditions using a linear mixed model. Based on the fecundity test results, the six pea genotypes were separated in two groups of resistant and susceptible groups against P123 and LSR1 (Fig. 1). The resistant pea genotypes to P123 were KAZAR, AeD3 and AeD4 and the susceptible ones were FP, AeD1 and AeD2; while the resistant pea genotypes to LSR1 were KAZAR, AeD3, AeD4 and AeD2 and the susceptible ones were AeD1 and FP (Fig. 1).

The statistical analysis revealed 64 DEGs between P123 resistant and susceptible pea genotypes (fdr_P_ResistanceP123 < 0.05, “fdr_P_ResistanceP123” means the FDR adjusted *p*-value of the statistical test of the comparison of gene expression levels between resistant and susceptible pea genotypes) (Table S7). These genes were significantly differentially expressed regardless of timepoints and infestation conditions. No significant DEG was identified for other interactions. This means that no gene was identified as being differentially induced or suppressed between resistant and susceptible genotypes upon P123 infestation. We thus regarded these 64 genes as likely involved in constitutive resistance. Of these, 41 also responded to P123 infestation, despite being constitutively differentially expressed between resistant and susceptible genotypes (Table S3). Eight of these 41 genes were down-regulated during P123 infestation and only at 120 hpi; four of them were detected in one genotype, while the other four were detected in at least three genotypes. One gene (among the 41) was identified as up-regulated and down-regulated during P123 infestation for the pea genotypes AeD1 and AeD2, respectively. The 32 remaining genes were up-regulated during infestation with P123. In the list of 64 differentially expressed genes between resistant and susceptible pea genotypes, 25 of the genes were more expressed by the resistant pea genotypes compared to the susceptible ones, and no GO term was significantly enriched (p-adjusted values < 0.05). Thirty-nine of the genes were identified as more expressed by the susceptible pea genotypes compared to the resistant pea genotypes, and no significant GO term was associated with these genes (p-adjusted values < 0.05). The GO enrichment analysis did not reveal any significant GO term for the two lists of 64 (25 + 39) constitutively differentially expressed genes. None of the 64 significant genes were located in the *ApRVII* locus.

The five most significant genes (Pvalue_ResistanceP123) from the 64 DEGs (Table S7 and Figure S3) did not point out specific pathways or functions involved in resistance. Two genes (*Psat5g198720.1* (FC = 7.9, “Tubulin C-terminal domain”) and *MSTRG.6987.2* (FC = Infinite “Unknown”)) were identified as more expressed in the resistant pea genotypes compared to the susceptible ones. Three genes were identified as less expressed by the resistant pea genotypes: *Psat7g125280.1* (FC = 9.1, Thioredoxin M3), *Psat0s2771g0240.1* (FC = 3.0, glycosyl hydrolase 1 family), and *Psat7g125320.1* (FC = 9.8, Thioredoxin M3).

The same statistical analysis was conducted on samples infested or not with LSR1. The analyses showed 202 DEGs between LSR1 resistant and susceptible pea genotypes at each timepoint and under each infestation condition (fdr_P_ResistanceLSR1 < 0.05) (Table S8). For all genes, the change in expression level due to LSR1 infestation, between different timepoints, and due to LSR1 infestation at different timepoints was not significant for susceptible and resistant genotypes (interactions resistance: infestation, resistance: timepoint, and resistance: timepoint: infestation). This result indicated that no gene was differentially induced or suppressed between resistant and susceptible genotypes upon infestation. Since no significant interaction was identified, these 202 genes were differentially expressed between resistant and susceptible genotypes in a constitutive manner. Seventy-eight of these 202 genes have already been identified as differentially expressed upon LSR1 infestation compared to non-infested samples (Table S3). Ninety-seven of the 202 genes were more expressed by the resistant pea genotypes compared to the susceptible ones, and no GO term enrichment was found (p-adjusted values < 0.05). One hundred and five of the genes were more expressed in the susceptible compared to the resistant pea genotypes, and no GO term enrichment was found (p-adjusted values < 0.05).

Among these 202 significant genes associated with the resistance to LSR1, five genes were located in the *ApRVII* locus. Two of these genes, *Psat7g089640.1* and *Psat7g091320.1* that encode proteins of unknown functions, were more expressed in the resistant than the susceptible pea genotypes. The three other genes (*Psat7g091200.1*,* Psat7g088160.1*,* MSTRG.24664.1*) were less expressed in the resistant compared to the susceptible pea genotypes. *Psat7g091200.1* is predicted to encode a protein of unknown function, *Psat7g088160.1* is predicted to encode a polygalacturonase QRT3, and *MSTRG.24664.1* is predicted to encode a not well characterized protein domain (Table S8).

The five most significant genes (Pvalue_ResistanceLSR1) of the 202 identified differentially expressed genes (Table S8 and Figure S4) indicated their potential involvement in aphid resistance. Three of these genes were more expressed in the resistant compared to the susceptible pea genotypes (*Psat6g004000.1* (FC = 4.0, “disease resistance protein”), *Psat6g003920.1* (FC = 5.5, “disease resistance protein”) and *MSTRG.19265.1* (FC = 11.8, “Unknown”)). Additional homology analyses by Blast indicated homologies of *Psat6g004000.1* and *Psat6g003920.1* with several genes from *P. sativum*, *Vicia villosa* and *M. truncatula* that are predicted to encode RPP13-like proteins. Two genes were less expressed by the resistant compared to the susceptible pea genotypes (*Psat6g040680.1* (FC = 3.7, “Amino acid permease”) and *Psat0s1664g0080.1* (FC = 8.0, “Endoplasmin homolog”)).

## Discussion

### Both pea-adapted and pea non-adapted aphid clones induce immune responses and secondary metabolite synthesis pathways in pea

In this study, we examined the transcriptional responses of pea genotypes to pea-adapted and pea non-adapted clones of *A. pisum* to identify commonalities and differences of pea responses in host and non-host interactions. Six pea genotypes with different levels of resistance to the two pea aphid biotypes were selected to identify the pathways/genes that might be involved in the partial aphid resistance, especially those controlled by the *ApRVII* locus.

The PCA analysis of global gene expression revealed a highly genotype-specific expression pattern, with similar patterns observed within the same genetic groups. However, no clear association was found between global gene expression and aphid resistance phenotype, suggesting that the expression of only a small subset of genes may contribute to the variation in aphid resistance levels, or that resistance is not conferred by the transcription of certain genes. These findings suggest the limitations of using genetically diverse lines for dissecting resistance mechanisms. Employing isogenic lines in future studies may allow a more precise identification of transcriptomic changes associated with aphid resistance loci.

Pea responses to both, the pea-adapted clone P123 and the non-adapted clone LSR1 showed some similarities, but the transcriptional changes induced by P123 infestation were stronger. Stronger transcriptional responses during host interactions compared to non-host interactions were also observed in other studies, one based on *Brassica napus* and the other on *A. thaliana* infested with plant-adapted or non-adapted aphid species [8, 9]. These observations indicate the possibility that non-host resistance to aphids may involve preformed components that do not require transcriptional regulation, such as physical barriers and deterrent or toxic molecules, or it may involve post-transcriptional responses [8, 9].

Although the number of DEGs was not as high as in the host interaction, some DEGs were identified in the pea responses to LSR1. Most of these DEGs were also differentially expressed during the interaction with P123. The enrichment analyses of this category of genes indicated up-regulation of plant parasite response pathways and secondary metabolite biosynthetic processes in all the pea genotypes. In *A. thaliana*, such pathways have been shown to be triggered by the pattern-triggered immune response [56]. Therefore, these pea responses can be considered as a general pea immune response to *A. pisum* infestation, most likely triggered by aphid HAMPs released by both, pea-adapted and non-adapted aphid clones.

Metabolite biosynthetic processes, which can potentially act as a defence response, were also induced by both the pea-adapted and non-adapted aphids. In particular, the biosynthesis of phenylpropanoids, including flavonoids and isoflavonoids, was induced. The flavonoids and isoflavonoids have various functions in plants, such as signaling molecules, components of physical or chemical barriers, or toxic metabolites for parasites [57]. The feeding behavior of the pea biotype of *A. pisum* on pea leaves treated with ethanolic solutions containing specific flavonoids and isoflavonoids was examined in a previous study [58]. Kaempferol, a flavonoid, and daidzein, an isoflavonoid, negatively affected stylet access to phloem tissue [58]. These results indicate that the production of some flavonoid or isoflavonoid compounds in pea could have a repellent effect on certain biotypes of the pea aphid. This hypothesis is further supported in the literature. An untargeted metabolomic study of different host legumes before and after infestation with adapted and non-adapted pea aphid biotypes showed that the pea genotype produced specific compounds in larger quantities upon interaction with the non-adapted biotype than the adapted biotype. One of these compounds was predicted to be a flavonoid [59]. Another study on *Medicago sativa* L. showed an increase in the production of an isoflavone compound, the genistein, during the interaction with the non-adapted biotype of *A. pisum* (pea-adapted biotype) but not with the adapted biotype (alfalfa-adapted biotype) [60]. The addition of genistein to an artificial diet showed that this compound reduced the survival of the non-adapted biotype but not the adapted biotype [60], indicating the involvement of genistein in host specificity of the aphids. In contrast, a study on *M. sativa* with a pea aphid did not reveal major change in flavonoid content in response to aphid infestation with the notable exception of a feruloyl conjugated flavone [61]. While the precise aphid biotype was not specified in the study, the relatively short period of phloem ingestion, the long period of non-probing and the fecundity suggest that this biotype is likely not adapted to *M. sativa*.

Despite the induction of the phenylpropanoid synthesis pathway, the pea-adapted aphids continued to thrive on the pea genotypes, suggesting that the pea-adapted aphids are not affected by the induction of the pathway. The pea-adapted aphids may be able to detoxify or sequester the produced phenylpropanoids. On the other hand, it is likely that the pea-non-adapted aphid suffered from the specific phenylpropanoids produced during the infestation, whereas pea-adapted aphids can tolerate these compounds. Despite the lack of evidence at a transcriptional level, it remains possible that the pea adapted aphids might manipulate the phenylpropanoid production by interfering with post-transcriptional processes using secreted salivary effectors.

### Global pea expression patterns during aphid infestation indicated a major transcriptional reprograming induced by the pea-adapted clone

Various biological processes were down-regulated in pea during infestation with P123, notably the photosynthetic processes for which a significant number of genes had their expression repressed. Several studies have shown that pathogen-associated molecular patterns (PAMP)-triggered immunity (PTI) leads to the down-regulation of photosynthesis associated processes [56, 62, 63]. A similar scenario may apply to herbivore-associated molecular pattern (HAMP)-triggered immunity in pea during aphid infestation.

In addition to the photosynthetic processes, the down-regulation of pathways involved in the biogenesis of cell wall compounds, fatty acid metabolism and various growth-related processes were observed. The down-regulation of these pathways may be a consequence of active reallocation of resources to enforce defence reactions, a survival strategy under nutrient-limiting conditions. The pea adapted clone feeds well on the peas and takes nutrients from the phloem sap, while the pea-non-adapted clone struggles to establish a prolonged feeding [20, 64]. Therefore, it is possible that the pea genotypes showed the transcriptional reprograming during the feeding by pea-adapted aphids but not by the non-adapted aphids. In parallel, we hypothesize that the down-regulation of various pathways may be a consequence of manipulation by the aphid effectors. Previous studies have suggested that the salivary protein composition differs between the pea and alfalfa biotypes of *A. pisum* [65]. In addition, infestation by the *A. pisum* pea biotype has been shown to induce susceptibility in pea to the alfalfa biotype [64], suggesting that specific salivary effectors of the pea biotype may actively manipulate pea immunity. For example, salivary effector of *R. padi* Rp1 suppresses expression of hormone signaling related genes in barley [66] and salivary effector of *Schizaphis graminum* Rondani Sg2204 suppresses PTI-induced callose deposition and jasmonic acid and salicylic acid associated defense gene expression in wheat [66, 67]. Some of the down-regulated pathways could play a role in inducing susceptibility in pea. We hypothesize that the fatty acid metabolism pathway may not only contribute to the biogenesis of cell wall components, but also to signaling during biotic interactions. Studies have highlighted the role of very-long-chain fatty acids and lipids in plant responses to pathogens [68]. In aphid-resistant soybean (*Glycine max*) genotypes, pathways involved in secondary cell wall compound production, including lignin biosynthesis, were shown to be activated as part of the defence response to *Aphis glycines* Matsumara [69]. Similarly, the lignin biosynthesis pathway has been implicated in the resistance of chrysanthemum to the aphid *Macrosiphoniella sanbourni* Gillette [70–72], indicating the importance of this pathway.

### Potential molecular mechanisms of *ApRVII*-mediated resistance to *A. pisum*

The pea genotypes carrying resistance-associated haplotypes at the *ApRVII* locus and showing resistant phenotypes to pea-adapted or pea-non-adapted genotypes did not show visible hypersensitive response or particularly stronger transcriptional responses compared to the susceptible genotypes. Other studies of legume-aphid interactions report different results. Genotypes of *M. truncatula* that contain resistance loci to the bluegreen aphid (*Acyrthosiphon kondoi* Shinji) or the pea aphid (*A. pisum*) show a higher expression of transcripts encoding specific transcription factors when infested by these aphids, compared to susceptible genotypes [73]. A transcriptomic analysis of resistant and susceptible *M. truncatula* near isogenic lines infested with spotted alfalfa aphids (*Therioaphis trifolii* (Monell) f. *maculata*) at 12 and 24 hpi showed enrichment of the genes involved in defense and stress responses in the resistant line [11, 74]. Furthermore, Lee et al. investigated the transcriptional response to soybean aphid (*A. glycines* (L.) Merr) colonization in soybean, at earlier time points (6, 12, and 48 hpi), comparing resistant and susceptible NILs differing at the *Rag5* locus [75]. Many DEGs were shared between the two genotypes and were predicted to be involved in defense-related pathways. However, the resistant NIL exhibited a more rapid response compared to the susceptible line. Overall, multiple comparative transcriptomic studies in legume species have shown that resistant genotypes carrying characterized resistance loci tend to display stronger or faster transcriptional responses than susceptible genotypes during the early stages of aphid infestation. In contrast, such enhanced transcriptional responses were not observed in the resistant pea genotypes used in our study, and their responses were also different from the case of cowpea and *A. craccivora* interaction where resistant line showed slower transcriptional responses to the aphid infestation. These differences may reflect variation in the nature or function of resistance genes across legume species. Whereas resistance loci in *Medicago* and *Glycine* appear to mediate strong early defense signaling, possibly through gene-for-gene recognition and rapid activation of downstream pathways, *ApRVII* in pea may function differently.

Although the involvement of non-transcriptional mechanisms in the *ApRVII*-mediated resistance is possible, here we hypothesized that small sets of differentially regulated genes may be involved in the *ApRVII*-mediated resistance to the aphid clones and identified the DEGs between resistant and susceptible genotypes. The expression levels of all identified DEGs differed constitutively between resistant and susceptible genotypes, suggesting the involvement of a constitutive component in *ApRVII*-mediated resistance.

Regarding resistance to the pea-adapted aphid clone P123, 64 genes were identified as differentially expressed between resistant and susceptible pea genotypes. Although statistically most significant genes did not show clear functions or pathways involved in aphid resistance, the list of 64 genes contained three interesting genes: two encoding peroxidases (*Psat1g168960.1* and *Psat5g250400.1*) and a third gene encoding the broad-spectrum resistance protein RPW8 (*Psat6g011280.1*). These genes were more expressed in resistant genotypes compared to susceptible ones. Peroxidases are known to play a role in the production of reactive oxygen species, which are involved in defence signaling and can also exert direct toxic effects on parasites [76]. RPW8 proteins are known to be involved in the recognition of biotrophic pathogens such as the powdery mildew, leading to hypersensitive responses [77].

Concerning the resistance to the pea non-adapted clone LSR1, 202 candidate genes were identified as differentially expressed between resistant and susceptible genotypes. Among these, two “disease resistance genes”—*Psat6g004000.1* and *Psat6g003920.1*—were identified as encoding RPP13-like proteins, a typical class of CC-NB-LRR receptor-like proteins [78], but they were not encoded at the *ApRVII* locus. These genes were more highly expressed in resistant than in susceptible pea genotypes. To understand the mechanisms of *ApRVII-*mediated resistance to *A. pisum* clones, it is necessary to examine the contribution of those differentially expressed genes on *A. pisum* resistance and understand how *ApRVII* affects their expression patterns.

A feeding study of pea-adapted and alfalfa-adapted *A. pisum* biotypes on five *P. sativum* genotypes with different resistance levels showed that higher pea resistance did not affect the feeding behavior of the pea-adapted biotype, indicating that the quality of the phloem sap of the resistant genotype is lower than that of susceptible genotype and reduces aphid fecundity [20]. The alfalfa-adapted *A. pisum* biotype had difficulties reaching the phloem and establishing feeding in the resistant pea genotypes. It is not known whether the molecules that affect the quality of the phloem and those that affect the establishment of phloem feeding of the pea-non-adapted aphid are the same or not, and whether the identified differentially expressed genes affect the production of such metabolites. Metabolomic studies of the pea genotypes and bioassays with the resistance-associated molecules may further contribute to the understanding of the mechanisms of *ApRVII-*mediated defence against the *A. pisum* biotypes. Pea mutant collections are available in some pea genotypes [79] and CRISPR/Cas9 mediated gene editing methods have been recently developed for pea [80–82]. Identification or creation of mutants of the candidate genes involved in aphid resistance will be an important next step to examine the molecular mechanisms of pea resistance to the aphid. Furthermore, identification and characterization of aphid salivary effectors will reveal the mechanism of pea manipulation by *A. pisum* pea biotype and provide important knowledge to create the pea genotypes that are resistant to the manipulation by the aphids.

## Conclusion

This study revealed the transcriptional responses of pea genotypes to both pea-adapted and non-adapted clones of *A. pisum*, highlighting key similarities and differences in these responses. Our findings indicate that pea transcriptional changes to the pea-adapted aphids are generally stronger than those to the pea-non adapted aphids, with global gene expression patterns being highly genotype-specific, but not directly correlated with aphid resistance phenotypes conferred by the *ApRVII* locus.

Both aphid biotypes are likely to trigger immune responses and secondary metabolite synthesis in pea, with flavonoid- and isoflavonoid-synthesis related pathways playing a potential role in resistance. In particular, flavonoid and isoflavonoid compounds may act as deterrents, especially against the non-adapted aphid biotype. The pea-adapted aphids appeared to suppress various pathways. We speculate that the suppression may be due to resource reallocation by pea or manipulation by aphid salivary effectors.

This study also identified candidate genes potentially involved in aphid resistance both within and outside of the *ApRVII* locus. However, the functions of most of the candidate genes remain uncharacterized and require further studies, including the creation and characterization of mutants of the candidate genes and their metabolomic analyses.

## Electronic supplementary material

Below is the link to the electronic supplementary material.

Supplementary Material 1

Supplementary Material 2

## Data Availability

The raw RNA sequence data reported in this manuscript have been deposited in NCBI as BioProject: PRJNA1228675.

## Abbreviations

DEG: Differentially expressed genes
FC: Fold change
GO: Gene ontology
GWAS: Genome-wide association study
HAMPs: Herbivore-associated molecular patterns
Hpi: Hours post-infestation
KEGG: Kyoto Encyclopedia of Genes and Genomes
NILs: Near isogenic lines
ORF: Open reading frame
QTL: Quantitative trait locus
