# Supplementary material for: Transcriptomic analysis reveals candidate molecular pathways involved in pea (Pisum sativum L.) resistance to pea aphid (Acyrthosiphon pisum Harris) biotypes
Source: BMC Genomics. 2025 Jul 1;26:580. doi: 10.1186/s12864-025-11742-3 (PMC12211313; doi:10.1186/s12864-025-11742-3)
Supplement: Supplementary file 2 — Supplementary Material 2 [file 12864_2025_11742_MOESM2_ESM.docx]

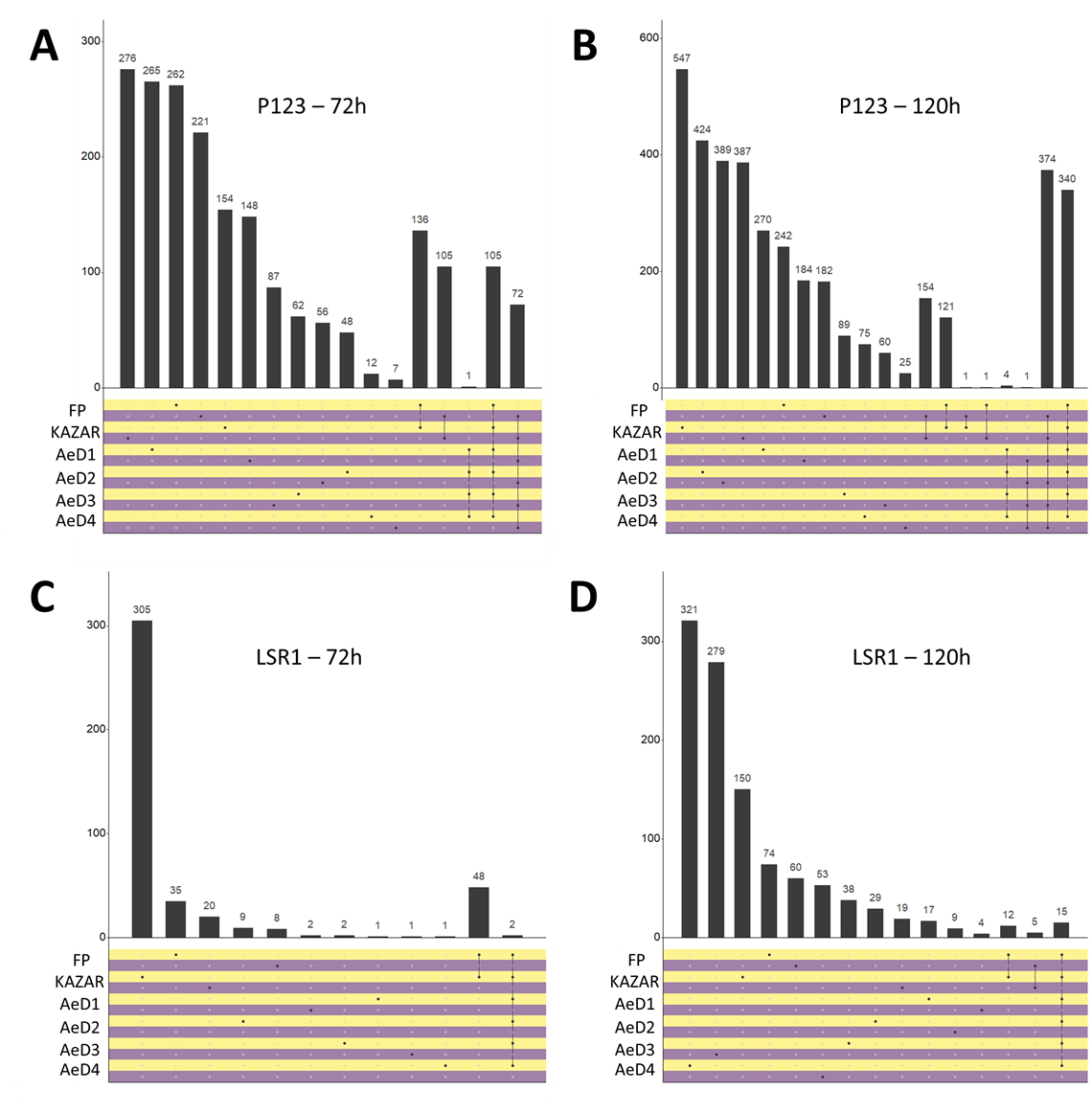


Figure S1: Intersections of differentially expressed genes (DEGs) lists. The DEGs are either up- (yellow stripes at bottom) or down-regulated (purple stripes) between different pea genotypes under different infestation conditions. Infestation with (A) P123 72 hpi, (B) P123 120 hpi, (C) LSR1 72 hpi (D) LSR1 120 hpi.


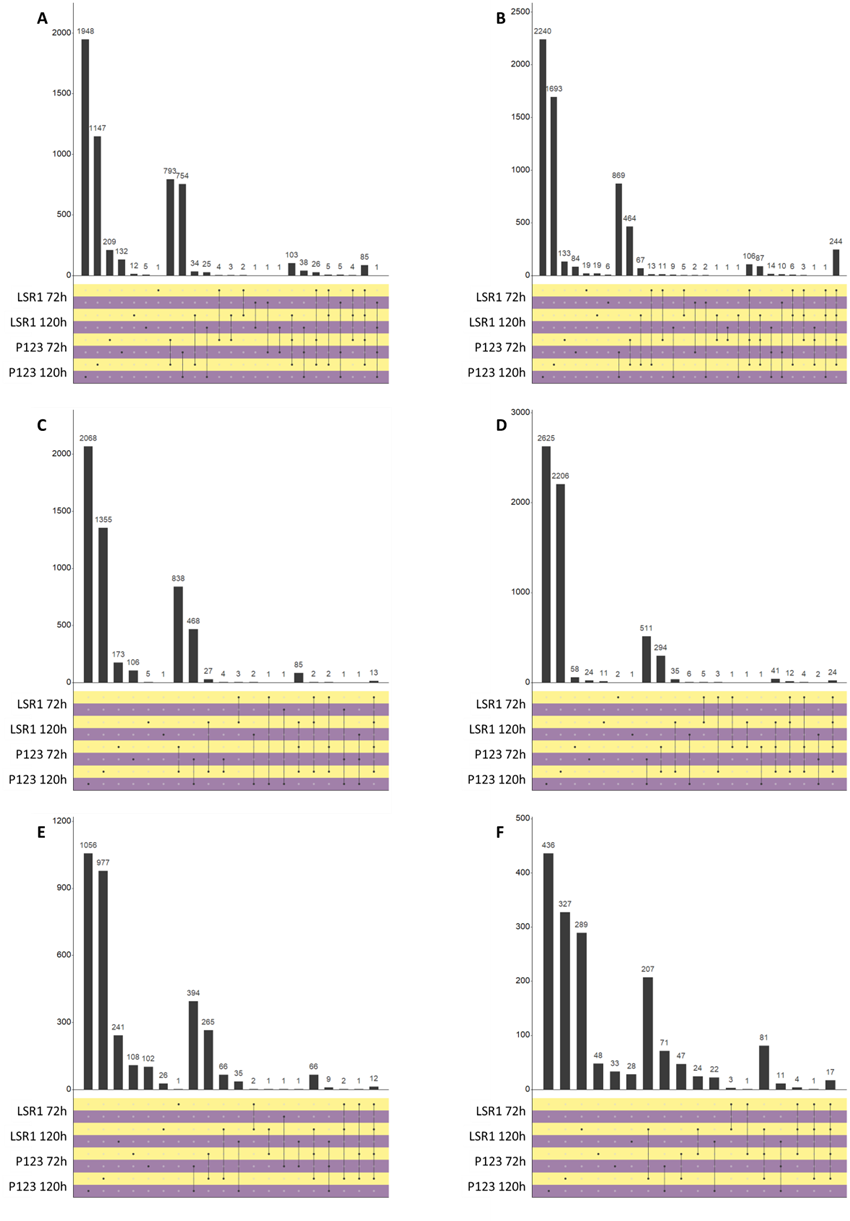


Figure S2: Intersections of differentially expressed genes (DEGs) lists. The DEGs are either up- (yellow bars at bottom) or down-regulated (purple bars) during infestation with P123 and LSR1 for the genotypes (A) FP, (B) KAZAR, (C) AeD1, (D) AeD2, (E) AeD3, (F) AeD4.


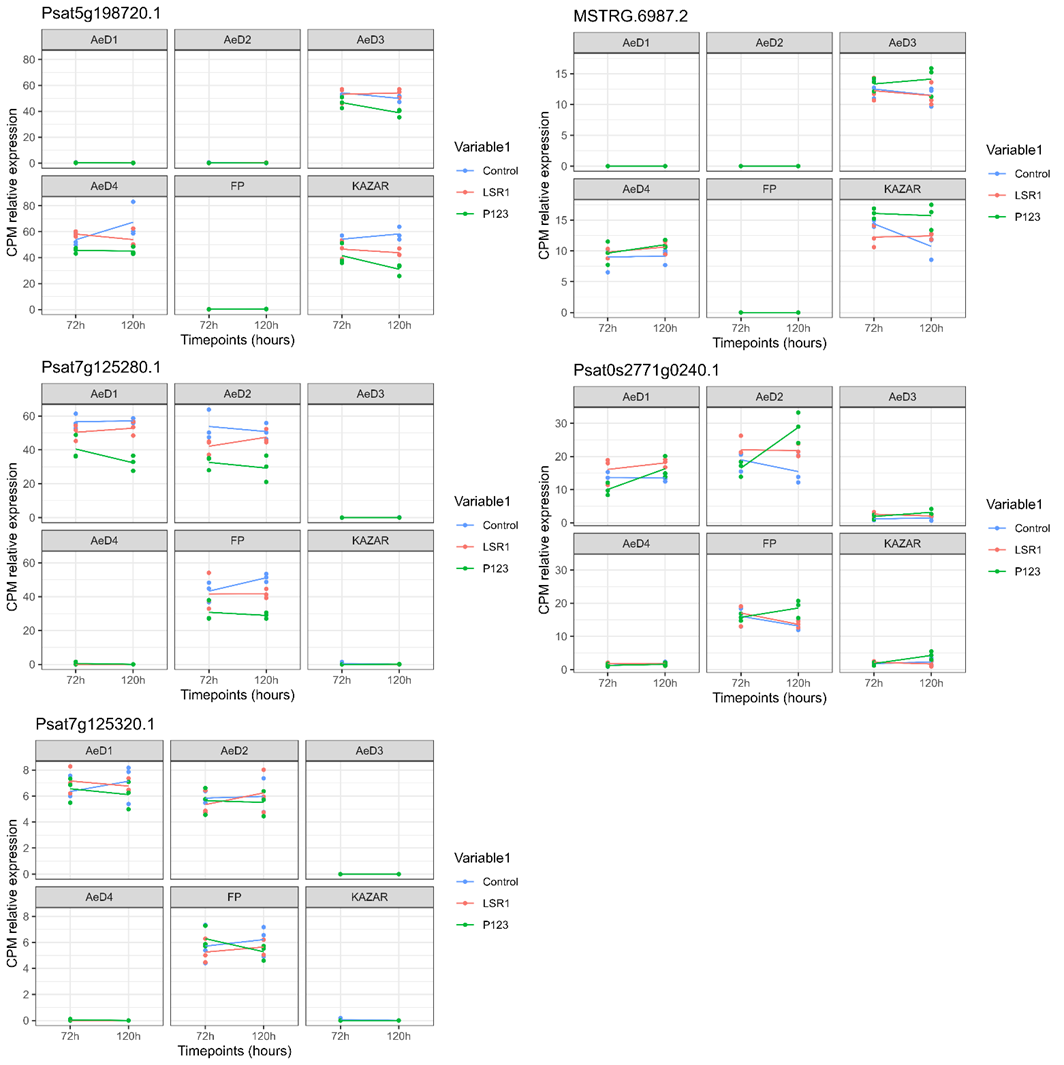


Figure S3: Relative expression of top five DEGs in pea genotypes for the resistance to P123. Relative expression (CPM: counts per million) of the five genes most significantly differentially expressed between resistant and susceptible pea genotypes to the pea-adapted clone P123.


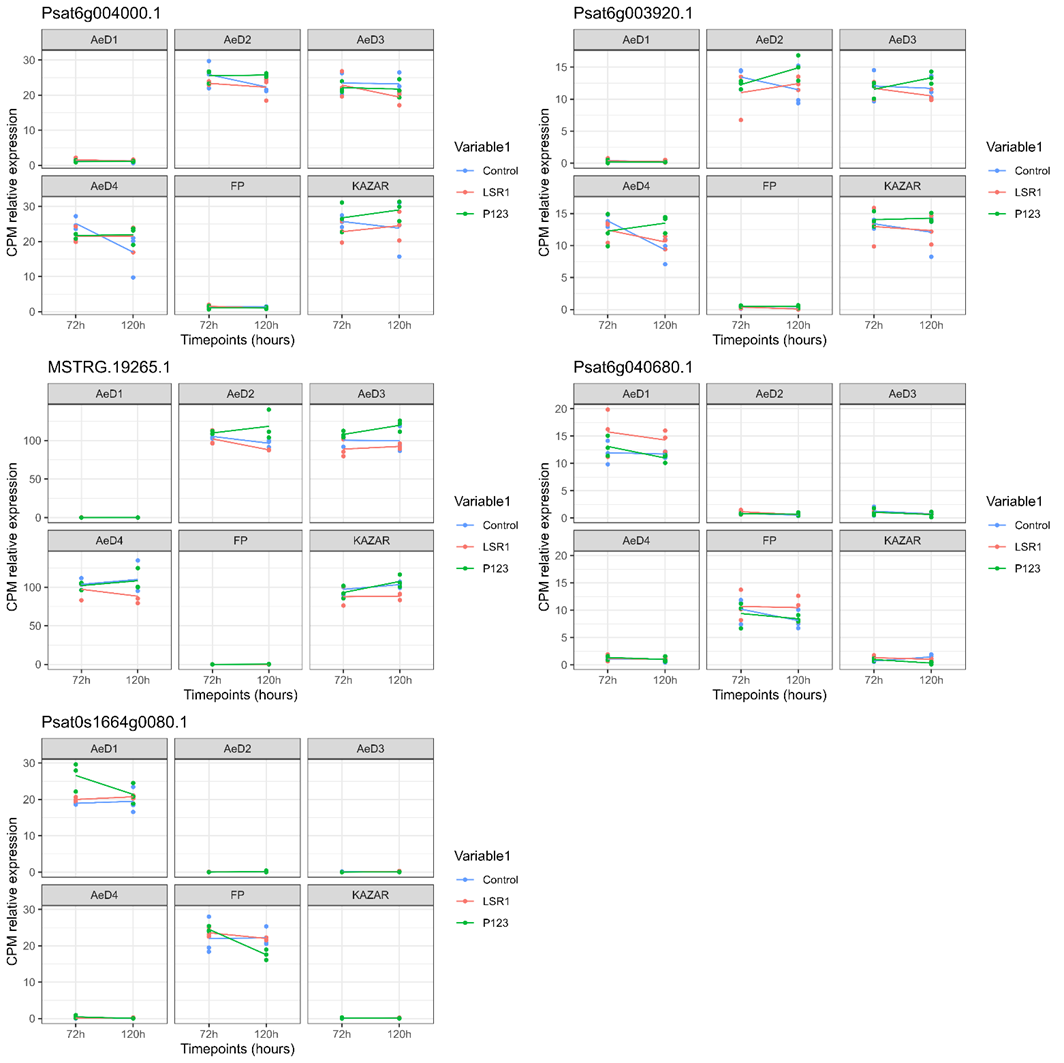


Figure S4: Relative expression of top five DEGs in pea genotypes for the resistance to LSR1. Relative expression (CPM: counts per million) of the five genes most significantly differentially expressed between resistant and susceptible pea genotypes to the pea-adapted clone LSR1.
